# Epidemiology, bacteriology, and clinical characteristics of HACEK bacteremia and endocarditis: a population-based retrospective study

**DOI:** 10.1007/s10096-020-04035-y

**Published:** 2020-09-18

**Authors:** Andreas Berge, Christian Morenius, Alexandros Petropoulos, Bo Nilson, Magnus Rasmussen

**Affiliations:** 1 Unit of Infectious Diseases, Department of Medicine, Solna, Karolinska Institutet, Stockholm, Sweden; 2 Department of Infectious Diseases, Karolinska University Hospital, Stockholm, Sweden; 3 Department of Clinical Sciences Lund, Division of Infection Medicine, Lund University, Lund, Sweden; 4 Department of Microbiology, Tumor and Cell Biology, Karolinska Institutet, Stockholm, Sweden; 5 Department of Clinical microbiology, Karolinska University Hospital, Stockholm, Sweden; 6 Department of Laboratory Medicine Lund, Section of Medical Microbiology, Lund University, Lund, Sweden; 7 Clinical Microbiology, Labmedicin, Region Skåne, Lund, Sweden; 8 Division for Infectious Diseases, Skåne University Hospital, Lund, Sweden

**Keywords:** Endocarditis, Bacteremia, HACEK, Epidemiology, Echocardiography, Management score

## Abstract

The objective was to describe the epidemiology, bacteriology, clinical presentation, risk factors for endocarditis (IE), diagnostic workup, and outcome of patients with bacteremia caused by the non-*influenzae Haemophilus*, *Aggregatibacter*, *Cardiobacterium*, *Eikenella*, and *Kingella* genera (HACEK). A retrospective population-based cohort of patients with bacteremia collected from 2012 to 2017 was identified. Clinical data from identified patients were collected from medical records to classify patients, calculate incidences, analyze risk factors of IE, and describe the management and outcome of the cohort. A total of 118 episodes of HACEK bacteremia were identified, of which 27 were definite IE. The incidence of HACEK bacteremia was 5.2 and of HACEK IE 1.2 episodes per 1,000,000 inhabitants per year. Other focal infections were identified in 55 of 118 of the episodes, most commonly within the abdomen (26 episodes). The propensity to cause IE ranged from 62 in *Aggregatibacter actinomycetemcomitans* to 6% in *Eikenella*. Risk factors for IE were cardiac implantable electronical device, predisposing cardiac conditions, community acquisition, long duration of symptoms, multiple positive blood cultures, fever, heart murmur, embolization, and unknown origin of infection. The scoring system DENOVA developed to predict IE in bacteremia with *Enterococcus faecalis* also had a high sensitivity and specificity for predicting IE in HACEK bacteremia. The 30-day mortality was 4% in IE and 15% in non-IE bacteremia, and only one case of relapse was found. IE is common in bacteremia with *Aggregatibacter*, *Cardiobacterium*, and *Kingella* but relatively rare in *Haemophilus* and *Eikenella*. Treatment failures are very rare, and DENOVA can be used to evaluate the need for transesophageal echocardiography.

**Electronic supplementary material:**

The online version of this article (10.1007/s10096-020-04035-y) contains supplementary material, which is available to authorized users.

## Introduction

Bacteremia constitutes a clinical situation with a risk for different complications, of which infective endocarditis (IE) is of high relevance [1]. Faced with a positive blood culture (BC) result, the clinician has to evaluate the risk of IE and decide on the diagnostic workup and treatment. For bacteria that are common causes of IE, risk stratification systems to determine the need for transesophageal echocardiography (TEE) in bacteremia have been developed [2–6].

The genera of *Haemophilus* (except *H. influenzae*), *Aggregatibacter*, *Cardiobacterium*, *Eikenella*, and *Kingella* (HACEK) make up a group of gram-negative bacteria which are commensals of the oral cavity and gastrointestinal tract and are known to cause IE [7–9]. In a large international study, HACEK bacteria caused 1.3% of IE cases [8]. Other clinical manifestations of the HACEK are species and genus specific but they also have some general features, resulting in dental infections and bacteremia with unknown origin [7]. The risk for IE in cases of HACEK bacteremia has been shown to vary for the different HACEK genera [10]. In a previous study, all cases (18/18) of *Aggregatibacter actinomycetemcomitans* bacteremia represented IE, whereas no case of IE (0/11) was observed in bacteremia with *Eikenella corrodens*. Furthermore, bacteremia with *Haemophilus parainfluenzae* (10/18), *Cardiobacterium* (7/8), *Kingella* (8/19), and other species of the *Aggregatibacter* genus (9/13) had a very high propensity to cause IE [10]. The annual incidence of bacteremia in that study was 2.2 per million and for IE 1.4, including both definite and possible IE in the cohort (Dr Murdoch, personal communication).

A population-based study from the Danish national microbiology database focused on the epidemiology of HACEK bacteremia [11], not specifying the clinical conditions. The annual incidence of bacteremia with HACEK was found to be of 4.4 per million population. Of 147 episodes detected, 55 *Haemophilus* species, 37 *Aggregatibacter*, 9 *Cardiobacterium*, 21 *Eikenella*, and 27 cases of *Kingella* were found [11]*.*

Cephalosporins are the primary treatment options in international guidelines [1, 12], but due to lower levels of resistance, the Swedish recommendations advocate ampicillin as the first choice and cefotaxime when resistance is demonstrated.

In this study, we assembled a large population-based cohort of patients with HACEK bacteremia and describe the epidemiology, clinical presentation, diagnostic workup, risk factors for IE, and outcome. We also investigate whether scoring systems to determine the need for echocardiography in patients with bacteremia caused by streptococci and enterococci were applicable also on patients with HACEK bacteremia.

## Material and methods

All consecutive BCs positive for the HACEK genera from January 2012 to December 2017 were obtained from the databases of the Clinical Microbiology Laboratory in Skåne County, (the only laboratory in the region with a catchment area of 1.3 million inhabitants and nine hospitals) and from Karolinska University Laboratory, Karolinska University Hospital, Stockholm, Sweden (analyzing BC from a population of 1.9 million inhabitants in the Stockholm County).

All medical records of patients with HACEK bacteremia were studied retrospectively. Ethical approval was obtained from the Ethics Committee of Lund University (2017/1002) and from the Ethics Committee review board in Stockholm (recordal 2015/1184-31).

### Microbiology

#### Blood culture system and incubation

The BacT/Alert culture media (aerobic, anaerobic, and pediatric) and BacT/Alert 3D incubator (bioMérieux, Durham, NC, USA) according to the manufacturer’s instructions were used during the whole study period for BC in Stockholm County. In the Skåne County, the same BacT/Alert blood culture system (bioMérieux, Durham, NC, USA) was used from 2012 to late 2014, and it was replaced by the BACTEC FX blood culture system (Becton Dickinson, Franklin Lakes, NJ, USA) using the BD BACTEC culture media (Plus Aerobic/F, Lytic/10 Anaerobic and Peds Plus/F) in December 2014, and it was used for the remaining period of the study.

#### Species identification

Determination of genera and species was performed with Microflex matrix-assisted laser desorption/ionization time-of-flight (MALDI-TOF) mass spectrometry (MS) at both centers, using the direct transfer method as described previously [13]. The generated mass spectra of the bacterial isolates were analyzed with the MALDI Biotyper 4.1 software and the MALDI Biotyper Library DB-7854 (Bruker, Bremen, Germany). If a MALDI Biotyper score value of ≥ 2.0 was obtained in the routine analysis, this was considered to be reliable to the species level. In cases where a score of < 2.0 and ≥ 1.8 was found and the second-best species had a score difference greater than 0.2, the identification of the species was considered reliable as previously described [14]. In other cases, a new MALDI-TOF MS analysis was made on available stored isolates using the standard ethanol–formic acid extraction method described by the instrument manufacturer (Bruker, Bremen, Germany). The remaining isolates were analyzed by sequencing the 16S rRNA gene and assigned a species at the two centers as described [15, 16] when retrievable. In two isolates (one isolate of *Haemophilus* and one of *Eikenella*), the species determination was not successful by neither MALDI-TOF nor 16S, and the isolates were assigned only to the genus level.

#### Antimicrobial susceptibility testing

Antimicrobial susceptibility testing was performed at the clinical microbiology laboratories according to the EUCAST methodology for MIC determination using gradient tests (Etest, Oxoid) [17]. Müller–Hinton fastidious broth agar plates produced by the Substrate department at Karolinska University Hospital and by Clinical Microbiology, Labmedicin, Lund, were used. Bacterial inoculum of 0.5 McFarland and gradient test were applied on agar plates, and inhibition of growth was judged with naked eye after 18 h (± 2 h) of incubation in 35 °C (± 1 °C) in 4–6% CO_2_ environment.

### Definitions

An episode of HACEK bacteremia was defined as a clinical situation in a patient resulting in BC taken by the decision of the treating clinician, showing growth of a HACEK isolate. All BCs with growth of HACEK bacteria were defining an episode. No episodes were excluded, assuming the finding to be a contamination. Multiple positive BCs taken on different days were included in the same episode if they were taken during the same clinical situation. To be able to discriminate BCs taken within an episode from a new episode of bacteremia, an episode was delimited by at least 7 days of effective treatment or by 30 days.

IE was defined using the modified Duke criteria [18]. With regards to the Duke criteria, the HACEK bacteria were considered “a typical microorganism consistent with IE,” so growth in two separate blood cultures was sufficient to constitute a major criterion. In cases where the major Duke criterion for echocardiography was not met, the ESC criteria were used to further confirm the diagnosis of IE using ECG-triggered cardiac CT or ^18^FDG-PET-CT [1]. Other focal infections were diagnosed as described [2]. If two out of three of the following findings were found, a diagnosis was considered established: (1) typical signs or symptoms of the infection; (2) imaging results indicating the diagnosis; and (3) microbiological result, other than BC, confirming the diagnosis [2].

To evaluate the scoring systems NOVA, DENOVA, and HANDOC [2, 3, 5], scoring of patients was performed using the information available to the clinician at the time when receiving the positive BC result, analyzed to the genus or species. NOVA score parameters were defined as described by Bouza et al. [3] with modifications of number of cultures as described by Dahl et al. [19], and DENOVA and HANDOC according to the publications [2, 5]. An “etiology” of *Aggregatibacter*, *Cardiobacterium*, or *Kingella* was given one point for the A in the calculation of the HANDOC score [5]. A relapse was defined as a BC with growth of the same genus or species within the 365 days of follow-up after an episode. Comorbidities were classified according to the Charlson index [20].

### Data collection

Clinical data from each episode were collected from 365 days before its start until 365 days after the first positive BC. Thus, age, gender, comorbidities, previous bacteremia, symptoms, signs, performed radiology and its results, culture results other than blood cultures, hematuria, duration of symptoms, death within 365 days of positive culture, days hospitalized, and cultures or clinical conditions indicating therapeutic failure during follow-up were registered. Furthermore, data on intravenous drug use, other predisposing heart conditions, fever, vascular or immunological phenomena, microbiological data fulfilling or not fulfilling the prerequisites for Duke minor or major criteria, and whether transthoracic echocardiography (TTE) or TEE was performed and if diagnostic criteria for IE were met [1, 18] were collected. Missing data were registered as lack of result in that variable. No imputations were made.

### Statistics

The analysis of the collected data was calculated in the statistical program Stata (StataCorp, College Station, TX, USA). The odds ratios and their confidence intervals were calculated when applicable. The *χ*^2^ test was used when applicable, and otherwise, the *p* value of Fisher’s exact test was used. Differences between continuous variables were analyzed with Wilcoxon’s rank-sum test. Values are presented as proportions or medians with interquartile ranges (IQR).

## Results

### HACEK bacteremia

The cohort constituted 3.2 million inhabitants who were studied for 7 years, and 118 episodes of HACEK bacteremia were found, resulting in an incidence of 5.3 cases per 1,000,000 and year (Table 1). Most episodes were caused by *Haemophilus*, *Aggregatibacter*, and *Eikenella*, while fewer episodes were caused by *Cardiobacterium* and *Kingella* (Table 1). The occurrence of IE and other focal infections for the different species and genera is shown (Table 2). *Haemophilus* and *Eikenella* episodes often had abdominal origin of infection, and mainly *Haemophilus* were causing urinary tract infections. Infection foci other than IE, due to hematogenous seeding of bacteria, were mainly caused by the *Aggregatibacter* genus (3/4). All genera had 25% or more episodes with an unknown focus (Table 2).

**Table 1:** Cases of bacteremia and definite IE and the annual incidence (*n* per 1,000,000 inhabitants). Propensity of the genera and species to cause IE

Genus/species	Bacteremia, cases	Bacteremia, incidence	IE, cases (IE/B* (%))	IE, incidence
All HACEK episodes	118	5.3	27 (23)	1.2
*Haemophilus* species^	36	1.6	5 (14)	0.22
*H. parainfluenzae*	29	1.3	5 (17)	0.22
*H. haemolyticus*	1	0.04	0 (0)	0
*H. parahaemolyticus*	5	0.22	0 (0)	0
*Aggregatibacter* species	36	1.6	14 (39)	0.62
*A. actinomycetemcomitans*	13	0.58	8 (62)	0.36
*A. aphrophilus*	20	0.89	5 (25)	0.22
*A. segnis*	3	0.13	1 (33)	0.04
*Cardiobacterium hominis*	6	0.27	3 (50)	0.13
*Eikenella corrodens*	31	1.4	2 (6)	0.09
*Kingella kingae*	9	0.40	3 (33)	0.13

*B, bacteremia

^^^One BC isolate only assigned to the genus level, a dental infection

**Table 2:** Distribution of focal infections divided into genera and species

Genus/species	Bacteremia	IE	Unknown focus	Abdominal	Urinary tract	Wound	Oral	Respiratory	Joint	Spinal
All HACEK episodes	118	27	47	26	4	7	7	2	1	3
*Haemophilus* species^	36	5	14	9	3	2	3	0	0	0
*H. parainfluenzae*	29	5	14	4	2	2	2	0	0	0
*H. haemolyticus*	1	0	0	0	1	0	0	0	0	0
*H. parahaemolyticus*	5	0	0	5	0	0	0	0	0	0
*Aggregatibacter* species	36	14	13	3	0	3	2	2	1	2
*A. actinomycetemcomitans*	13	8	4	0	0	0	1	1	0	1
*A. aphrophilus*	20	5	7	3	0	3	1	1	1	1
*A. segnis*	3	1	2	0	0	0	0	0	0	0
*Cardiobacterium hominis*	6	3	3	0	0	0	0	0	0	1
*Eikenella corrodens*	31	2	12	14	1	1	2	0	0	0
*Kingella kingae*	9	3	5	0	0	1	0	0	0	0

^^^One BC isolate only assigned to the genus level, a dental infection

### IE caused by HACEK

In all, 27 cases of definite IE were identified, resulting in an incidence of 1.2/10^6^/year.

The IE episodes were 5, 14, 3, 2, and 3 in the five genera in HACEK, giving an incidence of 0.62 of *Aggregatibacter* IE, the genus most often causing IE (Table 1). All *Haemophilus* IE episodes were caused by *H. parainfluenzae*, and no cases of IE caused by *Haemophilus haemolyticus* or *Haemophilus parahaemolyticus* were found*.* The propensity to cause IE was very diverse among the genera and species (Table 1). The most prone genus to cause IE was the *Aggregatibacter*, *Cardiobacterium*, and *Kingella* genera (33-50% IE), while *Haemophilus* and *Eikenella* were less prone (14 and 6%). Among the species, *A. actinomycetemcomitans* was the most common cause of IE (8 episodes) and most prone to cause IE (62% of episodes) (Table 1). However, *A. actinomycetemcomitans* was the only species significantly more prone to cause IE compared with the other species in the cohort (Table 3).

**Table 3:** Propensity for genera and species to cause definite IE compared with the rest of the cohort

Pathogen	IE *n* = 27 (%)	Non-IE *n* = 91	Odds ratio (95% CI)	*P* value
*Haemophilus* species	5	31	0.44 (0.15–1.3)	0.12
*H. parainfluenzae*	5	24	0.63 (0.22–1.9)	0.41
*H. haemolyticus*	0	1	1.1 (0.04–28)	0.96
*H. parahaemolyticus*	0	5	0.29 (0.02–5.3)	0.29
*Aggregatibacter* species	14	22	3.4 (1.4–8.3)	*0.006*
*A. actinomycetemcomitans*	8	5	7.2 (2.1–25)	*0.001*
*A. aphrophilus*	5	15	1.1 (0.38–3.5)	0.80
*A. segnis*	1	2	1.7 (0.15–20)	0.67
*Cardiobacterium hominis*	3	3	3.7 (0.7–19)	0.13
*Eikenella corrodens*	2	29	0.17 (0.04–0.77)	*0.012*
*Kingella kingae*	3	6	1.8 (0.41–7.6)	0.43

Differences, calculated with the *χ*^2^ test when applicable and otherwise Fisher’s exact test, between the individual genus or species and the rest of the cohort are shown. Significant differences are shown in italics

### Comparison of episodes of HACEK bacteremia with and without IE

A comparison of clinical characteristics of episodes with HACEK IE and non-IE is shown in Table 4. Patients with HACEK IE were slightly older and had lower Charlson comorbidity score than patients without IE, but the differences were not significant. Furthermore, a long duration of symptoms, the presence of a cardiac implantable electronical device (CIED), prosthetic heart valve or native heart valve disease, heart murmur, fever, embolization, growth in all or the majority of BC, only one species in the BC, and unknown origin of infection were all significantly more common in patients with IE (Table 4).

**Table 4:** Characteristics of the cohort divided in episodes diagnosed with definite IE and without definite IE. Significant differences in favor of definite IE are shown in italics

Characteristics	IE (*n* = 27)	Non-IE (*n* = 91)	Odds ratio (95% CI)	*P* value
Age (median (IQR))	65 (52–72)	61 (44–76)	n/a	0.75
Sex (female)	9 (33)	32 (34)	0.92 (0.37–2.3)	0.86
Charlson score (median (IQR))	1 (0–2)	2 (0–3)	n/a	0.09
Community acquired	24 (89)	63 (72)	3.6 (0.98–13)	*0.042*
Health care associated	3 (11)	24 (26)	0.35 (0.10–1.3)	0.10
Nosocomial	0 (0)	4 (4)	1	0.57
Duration of symptoms (median (IQR))	15 (5–60)	3 (1–7)	n/a	*< 0.001*
Duration of symptoms ≥ 7 days*	18 (67)	24 (26)	5.6 (2.2–14)	*< 0.001*
CIED	6 (22)	3 (3)	8.4 (1.9–36)	*0.004*
Predisposition	15 (56)	15 (18)	5.9 (2.3–15)	*< 0.001*
Prosthetic valve	11 (46)	13 (14)	4.1 (1.6–11)	*0.005*
Native valve disease	5 (19)	2 (2)	10 (1.8–56)	*0.007*
Previous IE	1 (4)	2 (2)	1.7 (0.15–20)	0.54
Intravenous drug use	0 (0)	1 (1)	1	1.0
Prosthetic vascular graft	1 (3)	26 (5)	0.63 (0.08–4.8)	1.0
Heart murmur	9 (33)	7 (8)	6 (2.0–18)	*0.002*
Fever ≥ 38 degrees	26 (96)	66 (73)	9.8 (1.3–76)	*0.009*
Embolization	11 (41)	2 (2)	31 (6.2–151)	*< 0.001*
Number of positive cultures ≥ 2	23 (85)	44 (48)	6.1 (2.0–19)	*0.001*
Only one species in culture	27 (100)	54 (59)	n/a	*< 0.001*
Origin of infection, any	2 (7)	44 (48)	0.08 (0.02–0.38)	0.001
Respiratory tract	0 (0)	2 (2)	1	1.0
Gastrointestinal or biliary	0 (0)	26 (29)	1	0.002
Urinary tract infection	0 (0)	4 (4)	1	0.57
Tubo-ovarian abscess	0 (0)	1 (1)	1	1.0
Wound infection	0 (0)	7 (8)	1	0.35
Mediastinitis	1 (4)	0 (0)	n/a	0.23
Oral infection	2 (7)	5 (5)	1.4 (0.25–7.5)	0.66
Other focus	1 (4)	5(5)	1.3 (0.25–7.3)	0.73
Joint infection	0 (0)	1 (1)	1	1.0
Spondylodiscitis	1 (4)	2 (2)	1.7 (0.15–20)	0.54
Vascular graft infection	0 (0)	2 (2)	1	1.0
Unknown origin of infection	25 (93)	47 (52)	12 (2.6–52)	*< 0.001*

The odds ratios and their confidence intervals were calculated when applicable. The *χ*^2^ test was used when applicable and otherwise the *p* value of Fisher’s exact test. Continuous variables were analyzed with Wilcoxon’s rank-sum test

*Dichotomous variable used in the scores

### Possible IE

**Fig. 1:**
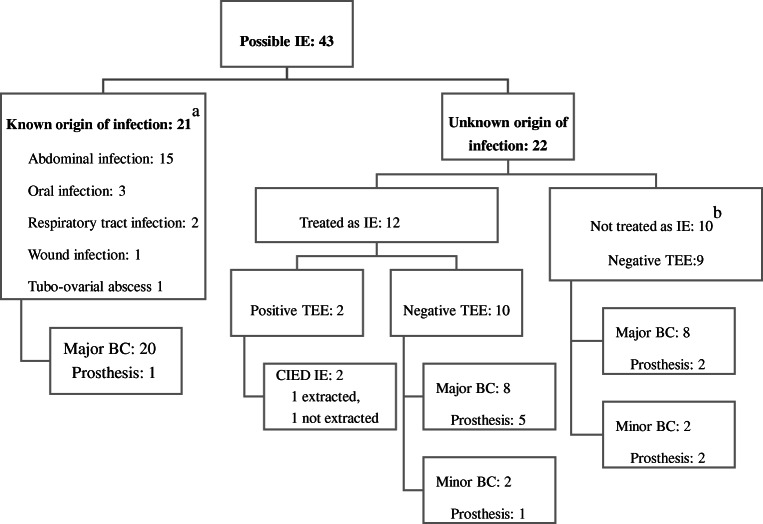
Description of episodes with possible IE based on clinical Duke criteria. ^a^One patient was diagnosed with two possible origins of infection during one episode. ^b^Includes two patients with spondylodiscitis, one with aortic graft infection, and one with septic arthritis. BC, blood culture. Major, major criterion in the Duke criteria. Minor, minor criterion in the Duke criteria. Prosthesis, valvular prosthesis

In addition to the 27 episodes of definite IE, 43 episodes fulfilled the clinical criteria for possible IE [18]. About half (21/43) of these were diagnosed with a known origin of infection, most commonly in the abdomen (Fig. 1). Eleven of these 21 were subjected to TTE or TEE (all negative) and had a median treatment time of 11 days (IQR 7–14). However, 22 had unknown origin of infection. Twelve of these 22 were treated as IE and 10 were not, with a median treatment time of 42 (IQR 31–44) and 13 (IQR 4–40), respectively. In six episodes treated as IE, a prosthetic valve was present, and in 4 episodes in the group not treated as IE (Fig. 1).

#### Susceptibility testing results

**Fig. 2:**
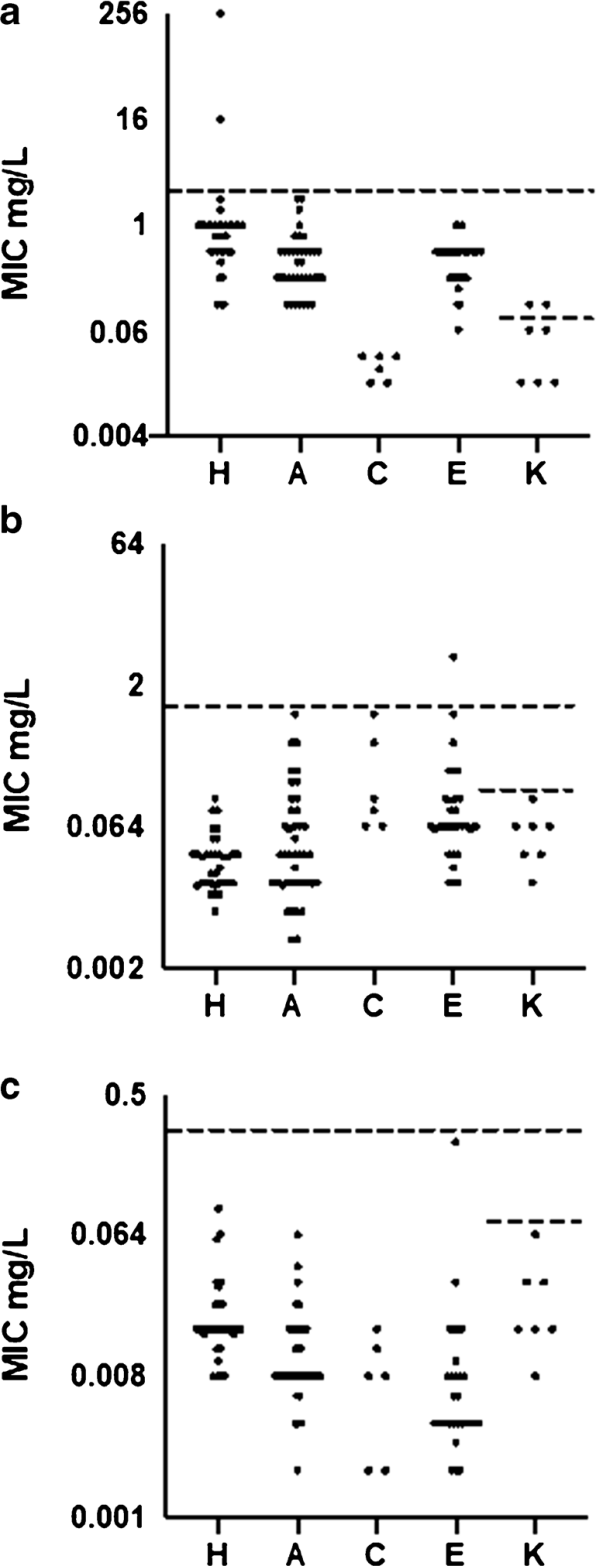
Distribution of MIC values for ampicillin (**a**), cefotaxime (**b**), and ciprofloxacin (**c**) for the isolates shown for each HACEK genus. The dashed lines represent the suggested cutoff, above which the isolate is regarded as resistant, according to EUCAST. The upper dashed line represents the EUCAST PK/PD breakpoints [21]. **a** Ampicillin 2 mg/L. **b** Cefotaxime 1 mg/L. **c** Ciprofloxacin 0.25 mg/L. The lower dashed line in column K represents the susceptibility break points for *Kingella* [22]. **a** Ampicillin 0.06 mg/L. **b** Cefotaxime 0.125 mg/L. **c** Ciprofloxacin 0.06 mg/L

Of 118 isolates tested for three antibiotics (ampicillin, cefotaxime, and ciprofloxacin), two had MIC > 2 mg/L for ampicillin, one had MIC > 1 mg/L for cefotaxime, and no isolate had MIC > 0.25 mg/L for ciprofloxacin (non-susceptible according to pharmacokinetic–pharmacodynamic breakpoints for these antibiotics [17]) (Fig. 2). However, according to the EUCAST epidemiological cutoff, two of the *Kingella* isolates were resistant to ampicillin using the cutoff at 0.06 mg/L [22] (Fig. 2).

### Performance of scoring systems

**Fig. 3:**
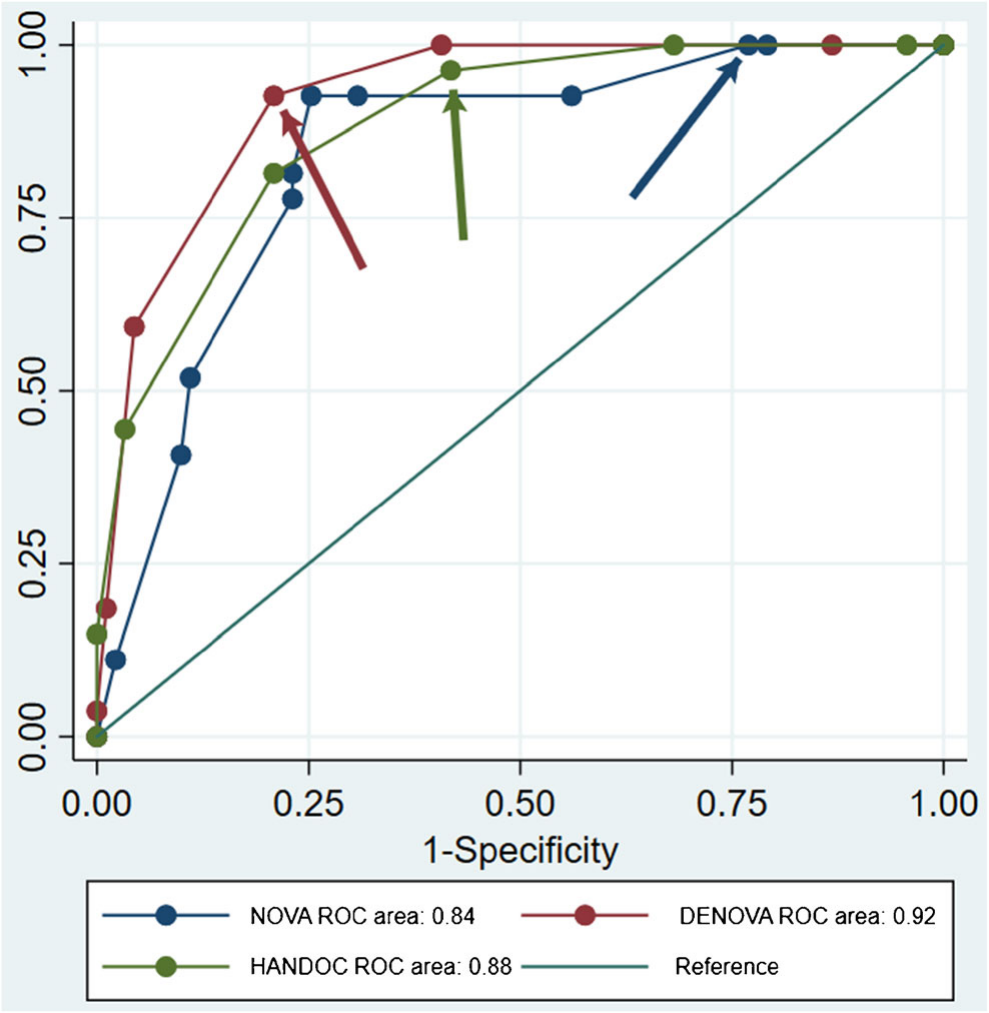
ROC curve for the scoring systems. Arrows in corresponding colors indicate the lowest score (4 for NOVA in blue, 3 for DENOVA in red, and 3 for HANDOC in green) for which each scoring system suggests TEE to be performed

The sensitivity and specificity to separate cases of HACEK IE from non-IE of the scoring systems, NOVA, DENOVA, and HANDOC, were tested. The data are shown in Supplementary table 1, and three ROC curves were constructed (Fig. 3). The area under the curve (AUC) for the three scoring systems was 0.84, 0.92, and 0.89, respectively. The DENOVA score had a significantly larger AUC than the NOVA score, and the AUC for the HANDOC score was intermediate (Supplementary table 1). At the predefined cutoffs, all scoring systems had a high sensitivity, ≥ 0.92 (Table 5), but the specificities were diverse, with 0.23, 0.79, and 0.49, respectively. That makes high negative predictive values for all scores but positive predictive value of 0.28, 0.57, and 0.36, respectively (Table 5).

**Table 5:** Characteristics of the scoring systems, NOVA, DENOVA, and HANDOC, to decide whether TEE can be omitted

Scoring system	Sensitivity (%)	Specificity (%)	PPV* (%)	NNS*	NPV* (%)
NOVA	100	23	28	3.6	100
DENOVA	93	79	57	1.8	98
HANDOC	96	49	36	2.8	99

*Positive predicted value (PPV), numbers needed to screen (NNS), and negative predictive value (NPV). The cutoff values set in the corresponding reports were used, NOVA ≥ 4, DENOVA ≥ 3, and HANDOC ≥ 3 [2, 3, 5]

### Treatment and investigations

The median treatment duration was 35 days in IE episodes and 12 days in non-IE (Table 6). In most episodes, beta-lactam antibiotics were chosen for the entire treatment time, but in 39 episodes (five with IE), the treatment was changed to ciprofloxacin. Ciprofloxacin was given for a median time of 10 days (IQR 5–17) in the whole cohort and for 38 days (IQR 28–60) in the IE episodes.

**Table 6:** Management and outcome of episodes of bacteremia with HACEK in the cohort

Management and outcome	IE (*n* = 27)	Non-IE (*n* = 91)	Odds ratio (95% CI)	*P* value
TTE performed	25 (93)	37 (41)	18 (4.0–82)	< 0.001
TEE performed	25 (93)	33 (36)	22 (4.9–99)	< 0.001
TTE or TEE performed	27 (100)	48 (53)	n/a	< 0.001
Heart surgery, incl. CIED extraction	13 (48)	0 (0)	n/a	< 0.001
Antibiotic treatment, days (median (IQR))	35 (25–46)	12 (5–17)	n/a	< 0.001
Days of hospital stay (median (IQR))	32 (25–39)	10 (5–17)	n/a	< 0.001
Relapse of infection	0	1*	1.4 (0.06–36)	0.83
Death within 30 days	1 (4)	14 (15)	0.21 (0.03–1.7)	0.19
Death during follow-up (365 days)	1 (4)	18 (20)	0.16 (0.02–1.2)	0.07

The odds ratios and their confidence intervals were calculated. The *χ*^2^ test was used when applicable and otherwise the *p* value of Fisher’s exact test. Continuous variables were analyzed with Wilcoxon’s rank-sum test

*See description of this case in the text

TTE or TEE was performed in all episodes of IE and in 53% of the non-IE episodes. TEE was done in 93% of IE episodes and 36% of non-IE episodes (Table 5). One patient was investigated using FDG-PET-CT, whereas no cardiac CT investigations were performed.

### Outcome

Only one relapse was identified during the follow-up time, and this was in a patient with growth of *Cardiobacterium* in one out of four BC bottles, prosthetic valve, fever, and a cerebral stroke that was suspected to be IE. The patient was subjected to transthoracic echocardiography, without IE findings, but not to TEE, and was given 4 days of ampicillin. After 180 days, the patient had a new stroke and was found to have IE.

The mortality, within 30 and 365 days, was lower in the patients with episodes of IE than in non-IE (4% and 4% compared with 15% and 20%, respectively), but the difference did not reach significance (Table 6).

Patients that died were significantly older, median 75 years (IQR 57–84), than the rest of the cohort, 60 years (IQR 39–73) (*p* value: 0.004), and had a higher Charlson score (median 4 (IQR 2–8) and 1 (IQR 0–2), respectively, *p* = value: < 0.001) (data not shown). The one diseased IE patient died of a massive intracranial hemorrhage. Thirteen patients had metastasized cancer or hematological cancer. Of the remaining five diseased patients, all died within 30 days. Three had polymicrobial infections, one was a 96-year-old woman diagnosed with pneumonia, one had an abdominal infection, and the third had a wound infection in connection with severe vascular insufficiency. Two patients had monomicrobial infections, one was a 91-year-old woman diagnosed with pneumonia after operation of a broken femur and one was admitted with massive intracranial hemorrhage and died within 2 days.

## Discussion

In this study, we describe the epidemiology, bacteriology, clinical presentation, and diagnostic workup of episodes of bacteremia of HACEK in a population-based cohort from two regions in Sweden. One of our main findings was that all the genera and species in the group had a high propensity to cause IE but that the inter-genus differences were large. This is in line with previous findings [10]. The genus of *Aggregatibacter*, especially the species *A. actinomycetemcomitans*, and *Cardiobacterium hominis* were most prone to cause IE, 62% and 50%, respectively (Table 1). These propensities were much higher than those of other species and genera also known to cause IE, for example 11% for *Staphylococcus aureus* [6], 12–13% for *Enterococcus faecalis* [19, 23], and 7.7% for *Viridans streptococci* [5].

Our study found a higher incidence of HACEK bacteremia, 5.3 episodes/10^6^/year, compared with the previous population-based studies [10, 11]. The incidences for the individual genera and species were similar, except for *A. actinomycetemcomitans* and *Eikenella* which were higher in our study compared with the Danish one [11]. We found an annual incidence of definite IE of 1.2 episodes/10^6^/year, which is similar to that described by Yew et al. [10]. However, in that study, both possible and definite IE were summed up to an incidence of 1.4. If the possible IE episodes in our study were added, the incidence of IE would rise to 2.2. Taken together, the three studies indicate a similar epidemiology with some local variations. Improved bacteriological diagnostics methods and an increased indication for performing blood cultures could contribute to the higher incidence in later studies.

There are no EUCAST species-specific breakpoints for HACEK bacteria except for *Kingella kingae* [22]. However, the interpretation of MIC values can instead be performed using the pharmacokinetic–pharmacodynamic breakpoints, or for *H. parainfluenzae*, *H. influenzae* breakpoints can be used [21]. Very few isolates in our study showed resistance to the antibiotics mainly used to treat HACEK infections. Analyzing our isolates, aware of the privileged Swedish context, there was no obvious ground to change the Swedish recommendation to use ampicillin as the empirical treatment after species determination. However, we found two isolates of *Kingella* with ampicillin MIC values higher than the EUCAST description of the wild type population, but lower than the pharmacokinetic–pharmacodynamic breakpoint of ampicillin [17, 21]. The lack of species-specific breakpoints makes it difficult to interpret resistance data reported for HACEK bacteria, and it would be valuable if such were developed for all HACEK species.

As expected, patients with IE had significant differences in underlying conditions and clinical presentation compared with the non-IE episodes. HACEK IE had similar risk factors as IE caused by streptococci, enterococci, and related genera [2, 5, 24]. Due to the limited number of IE cases and the high number of features associated with IE in univariable comparisons, we did not precede to make multivariable analyses. Thus, we cannot conclude which variables are independently associated with IE in HACEK bacteremia. Of interest, the presence of a CIED was correlated with IE, in contrast to multiple studies of bacterial species also known to cause IE [2, 3, 5, 24]. In fact, six out of 9 episodes of bacteremia in patients with CIED were diagnosed with IE. *Aggregatibacter* caused five of the six episodes of CIED-related IE, making a patient with *Aggregatibacter* bacteremia and a CIED highly likely to have IE.

When testing the scoring systems developed to estimate the risk of IE in bacteremia with other pathogens, DENOVA (developed for bacteremia with *E. faecalis*) performs best and could be used to guide the use of TEE in HACEK bacteremia. Interestingly, the two patients with IE not predicted by DENOVA to be at risk for IE had a CIED. Thus, we suggest that DENOVA could be used to guide the use of TEE in HACEK bacteremia but that TEE should always be performed in episodes of HACEK bacteremia in patients with CIED.

In our study, only one patient was subjected to FDG-PET-CT, a patient with *Aggregatibacter segnis* in blood cultures and an aortic graft infection. Thus, the ESC recommendations to proceed with PET-CT or cardiac CT after a negative TEE and continuous suspicion of IE [1] have not been used in our cohort. The only case reported as a relapse was likely an unfortunate missed case of IE, in whom TEE was not performed. We find it likely that the entire period of illness of this patient represents one infection episode with *Cardiobacterium*.

The international guidelines [1, 12] recommend antibiotic treatment for 4 weeks in native valve and 6 weeks in prosthetic valve HACEK IE. Many episodes with possible IE were treated with a shorter course than recommended for IE, with only one case of relapse. We therefore believe that cases of IE were not missed to any large extent. However, we cannot rule out that some case of missed IE was cured by a shorter course of antibiotics.

The retrospective design of our study limits the amount of information available for each patient. Only in 75 of 118 episodes a TTE or TEE was performed, and therefore, there is a risk that some episodes of IE might not have been detected. However, we consider that the number of missed IE episodes was limited because of the long follow-up period without relapse of bacteremia. Eighteen non-IE patients died during the follow-up, fourteen during the first 30 days from the positive blood culture, but only one arose suspicion of an undiagnosed IE. Only four patients died later than 30 days but within the year of observation. All these had malignancies, and all died without an apparent HACEK infection. It cannot be excluded that some of these had a relapse, though none of the deaths arose suspicion of undiagnosed infection according to our review of the medical records.

We believe that the present study contributes to the understanding of HACEK bacteremia and IE and how it should be managed. The clinician must have a high level of suspicion of IE in HACEK bacteremia, especially if caused by the genera *Aggregatibacter*, *Cardiobacterium*, and *Kingella*. The algorithm of DENOVA can provide additional aid in the decision in whether echocardiography should be performed.

## Electronic supplementary material

ESM 1 (DOCX 89 kb).

## Data Availability

The datasets analyzed during the current study are available from the corresponding author on reasonable request.
