# Supplementary material for: Epidemiology, bacteriology, and clinical characteristics of HACEK bacteremia and endocarditis: a population-based retrospective study
Source: Eur J Clin Microbiol Infect Dis. 2020 Sep 18;40(3):525–34. doi: 10.1007/s10096-020-04035-y (PMC7892745; doi:10.1007/s10096-020-04035-y)

Supplementary material

**Supplementary table 1**: The primary data represented as a ROC curve in Figure 3. The data for the different scoring systems are shown and an evaluation of the AUC of the individual curves and their confidence intervals. The cut-off specified in the corresponding publications are ≥ 4 for NOVA [3], ≥ 3 DENOVA [2], and ≥ 3 for HANDOC [5].


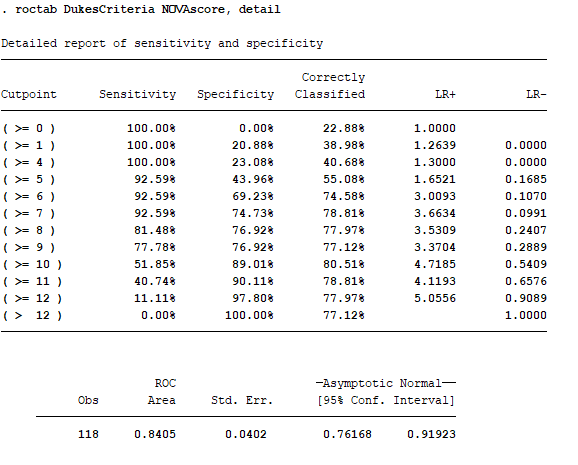


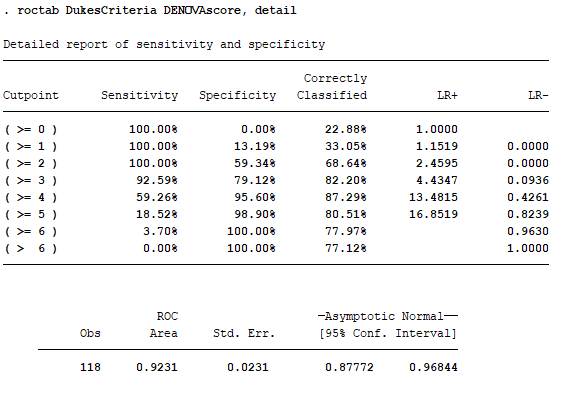


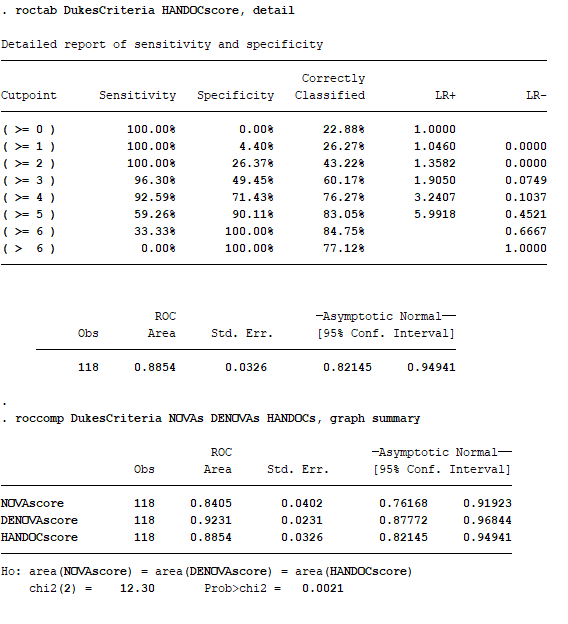

Supplement: Supplementary file 1 — (DOCX 89 kb). [file 10096_2020_4035_MOESM1_ESM.docx]
